# A Testis-Specific Long Noncoding RNA, *Start*, Is a Regulator of Steroidogenesis in Mouse Leydig Cells

**DOI:** 10.3389/fendo.2021.665874

**Published:** 2021-04-01

**Authors:** Kai Otsuka, Shin Matsubara, Akira Shiraishi, Natsumi Takei, Yui Satoh, Miho Terao, Shuji Takada, Tomoya Kotani, Honoo Satake, Atsushi P. Kimura

**Affiliations:** ^1^ Graduate School of Life Science, Hokkaido University, Sapporo, Japan; ^2^ Bioorganic Research Institute, Suntory Foundation for Life Sciences, Kyoto, Japan; ^3^ Department of Systems BioMedicine, National Research Institute for Child Health and Development, Tokyo, Japan; ^4^ Department of NCCHD Child Health and Development, Graduate School of Medical and Dental Sciences, Tokyo Medical and Dental University, Tokyo, Japan; ^5^ Department of Biological Sciences, Faculty of Science, Hokkaido University, Sapporo, Japan

**Keywords:** long noncoding RNA, spermatogenesis, testis, steroidogenesis, leydig cell, knockout mouse, testosterone

## Abstract

The testis expresses many long noncoding RNAs (lncRNAs), but their functions and overview of lncRNA variety are not well understood. The mouse *Prss/Tessp* locus contains six serine protease genes and two lncRNAs that have been suggested to play important roles in spermatogenesis. Here, we found a novel testis-specific lncRNA, *Start* (*Steroidogenesis activating lncRNA in testis*), in this locus. *Start* is 1822 nucleotides in length and was found to be localized mostly in the cytosol of germ cells and Leydig cells, although nuclear localization was also observed. *Start*-knockout (KO) mice generated by the CRISPR/Cas9 system were fertile and showed no morphological abnormality in adults. However, in adult *Start*-KO testes, RNA-seq and qRT-PCR analyses revealed an increase in the expression of steroidogenic genes such as *Star* and *Hsd3b1*, while ELISA analysis revealed that the testosterone levels in serum and testis were significantly low. Interestingly, at 8 days postpartum, both steroidogenic gene expression and testosterone level were decreased in *Start*-KO mice. Since overexpression of *Start* in two Leydig-derived cell lines resulted in elevation of the expression of steroidogenic genes including *Star* and *Hsd3b1*, *Start* is likely to be involved in their upregulation. The increase in expression of steroidogenic genes in adult *Start*-KO testes might be caused by a secondary effect *via* the androgen receptor autocrine pathway or the hypothalamus-pituitary-gonadal axis. Additionally, we observed a reduced number of Leydig cells at 8 days postpartum. Collectively, our results strongly suggest that *Start* is a regulator of steroidogenesis in Leydig cells. The current study provides an insight into the overall picture of the function of testis lncRNAs.

## Introduction

The testis has two main functions: spermatogenesis and androgen production. Spermatogenesis is regulated within the seminiferous tubules that contain germ cells and Sertoli cells and consists of three steps: mitosis, meiosis and spermiogenesis (1–3). Some of the mitotically proliferating spermatogonia enter meiosis to differentiate into haploid spermatids *via* primary and secondary spermatocytes, and mature spermatozoa are formed by spermiogenesis. During these processes, Sertoli cells nurse germ cells to support sperm development by releasing glial cell line-derived neurotrophic factor signaling molecules, retinoic acid, and growth factors (4–6). Leydig cells, another type of somatic cells, reside in the interstitial regions among the seminiferous tubules and produce androgens such as testosterone (7–9). Testosterone is a major androgen produced in the steroidogenesis pathway in Leydig cells and plays a critical role in spermatogenesis by targeting Sertoli cells (10, 11). Dysfunction of Leydig cells frequently leads to disruption of spermatogenesis due to insufficient intratesticular testosterone (12, 13).

In mammals, androgen production is precisely controlled by the hypothalamus-pituitary-gonadal (HPG) axis (14–16). The hypothalamus secretes gonadotropin-releasing hormone (GnRH) and induces the secretion of luteinizing hormone (LH) and follicle-stimulating hormone (FSH) from the pituitary to Leydig and Sertoli cells in the testis. Testosterone is produced and secreted in response to LH in Leydig cells for spermatogenesis and simultaneously suppresses the production and secretion of GnRH and gonadotropins in the hypothalamus and pituitary, respectively, by a negative feedback loop (15–17). Moreover, steroidogenesis is known to be controlled by autocrine and paracrine factors within the testis (18, 19). While the hormonal endocrine system that is responsible for steroidogenesis in the HPG axis has been relatively well characterized (20, 21), the molecular mechanism underlying the precise control of spermatogenetic genes is unclear. The findings in previous studies suggest some pivotal roles of a novel regulator in spermatogenesis and androgen production in the testis (22, 23).

Recent high-throughput studies have revealed that the testis expresses much more long noncoding RNAs (lncRNAs) than those expressed in other tissues (24, 25) and indicated differential expression of many lncRNAs in the testis (26, 27). lncRNAs are noncoding RNAs that are comprised of more than 200 nucleotides and they exhibit a wide variety of non-translational functions including gene regulation (28–32). During meiosis, the functions of several lncRNAs have been revealed. For instance, testis lncRNAs have been shown to function in meiotic entry in spermatogonia, activation of immune-related genes in primary spermatocytes, and regulation of postmeiotic genes (33–36). Moreover, some studies have suggested a function of lncRNAs in testicular somatic cells (37–40). However, the phenotype of knockout mice for testicular lncRNAs has been shown by only a few studies. Deletion of *Tsx*, an X-linked lncRNA, resulted in an increase in apoptotic cells due to meiotic arrest at pachynema, although mutant males were fertile (41). *Tslrn1*, a testis-specific lncRNA, was found to be involved in the formation of spermatozoa (42). In the case of *1700121C10Rik*, the knockout mice showed no obvious phenotype (43). These findings confirmed some physiological roles of the lncRNA in germ cells but results of *in vivo* evaluation of the functions of lncRNAs in Leydig cells have not been reported.

**Figure 1:**
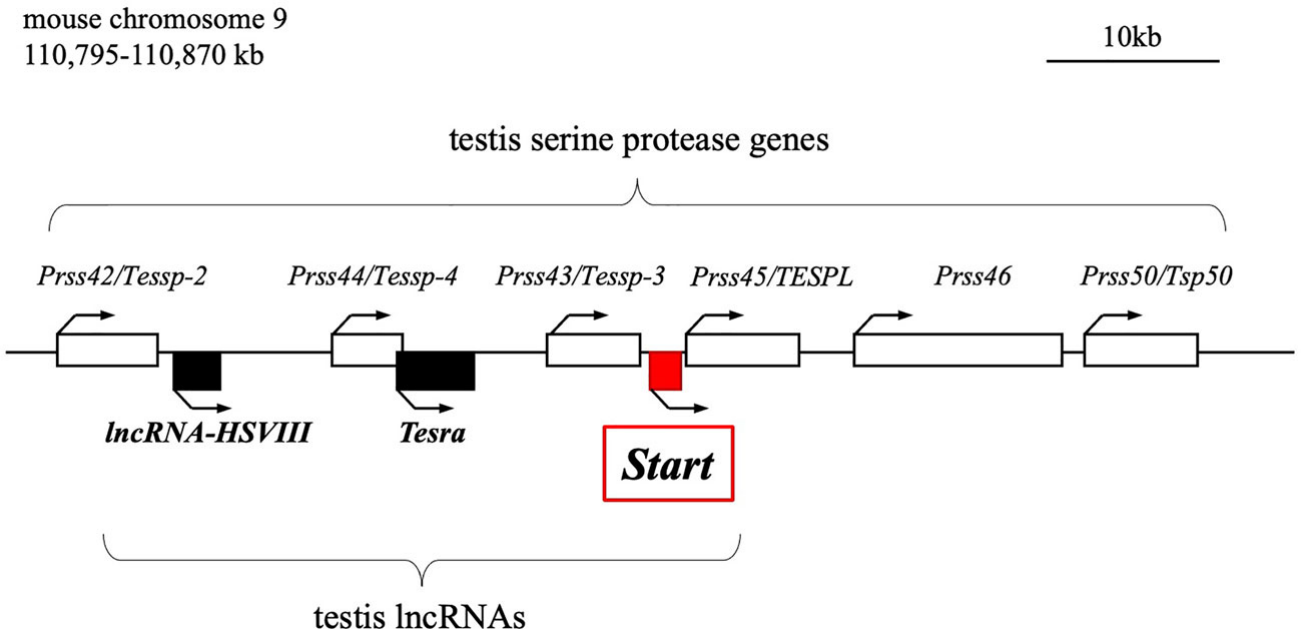
Schematic drawing of the mouse *Prss/Tessp* locus on chromosome 9. A 75-kb region corresponding to 110,795-110,870 kb of mouse chromosome 9 is shown. This region contains six genes encoding serine proteases and three lncRNAs as indicated by white and black/red boxes, respectively. *Start* is an lncRNA that was identified in this study and is indicated by a red-filled box. Bent arrows indicate the transcriptional direction of the protein-coding genes and lncRNAs.

We previously reported the identification of two testis-specific lncRNAs at the *Prss/Tessp* locus located on mouse chromosome 9F2-F3 (44, 45). This locus contains six *Prss* genes that are specifically or predominantly expressed in the testis and encode serine proteases (**Figure 1**) (46–48). Among the protein-coding genes, it has been suggested that *Prss42/Tessp-2* and *Prss43/Tessp-3* are essential for the progression of spermatogenesis and that the noncoding regions are involved in transcriptional regulation of the *Prss42/Tessp-2* gene (44–46). Those studies indicate that there are diverse molecular species and functions of lncRNAs in the testis. However, very little evidence for *in vivo* functions of lncRNAs in spermatogenesis and steroidogenesis has been provided.

In this study, we identified a novel lncRNA, *Start* (*Steroidogenesis activating lncRNA in testis*), transcribed at the *Prss/Tessp* locus. *Start* was found to be transcribed downstream of the *Prss43/Tessp-3* gene (**Figure 1**) exclusively in the testis, especially in germline and Leydig cells. Using *Start*-deficient mice and cultured Leydig cell lines, *Start* was found to be a regulator of steroidogenesis in the testis. This is the first identification of an lncRNA finetuning the steroidogenic pathway in the testis.

## Materials and Methods

### Animals

The experimental procedures used in this study were approved by the Institutional Animal Use and Care Committee at Hokkaido University and by the Animal Care and Use Committee of the National Research Institute for Child Health and Development. C57Bl/6Ncl mice (CLEA Japan Inc., Tokyo, Japan) and knockout (KO) mice (ICR and mixed background of C57Bl/6 and DBA2, Sankyo Labo Service Corporation) were maintained on 14 hr light/10 hr dark cycles at 25°C and given food and water ad libitum. In the analysis of *Start*-KO mice, we used male mice at 64-83 dpp as 2.5-month-old adult mice.

### Reverse Transcription-Polymerase Chain Reaction Analysis

Total RNAs were extracted with ISOGEN and ISOGEN II (Nippon Gene, Tokyo, Japan) for tissues and cultured cells, respectively, according to the manufacturer’s instructions. After treatment with TurboDNase (Thermo Fisher Scientific, Rockford, IL, USA), the RNAs were reverse-transcribed into cDNAs with the oligo(dT) primer or random hexamer using Superscript III (Invitrogen, Carlsbad, CA), according to the manufacturer’s instructions. A random hexamer was used for RT-PCR to check tissue distribution, and oligo(dT) was used for other experiments. PCR was performed using ExTaq polymerase (Takara, Otsu, Japan) or KOD Fx Neo (Toyobo, Tokyo, Japan). For strand-specific RT, “*Start* full length” reverse and “*Start* full length” forward primers were used to synthesize *Start* sense and antisense cDNAs, respectively, and a reaction temperature of 55°C was used to avoid non-specific binding of primers. Primer sequences are shown in **Table 1**. We repeated experiments with three or more biological replicates to confirm the reproducibility and showed a representative result.

**Table 1:** Oligo DNAs used in this study.

Name	Forward	Reverse
[RT-PCR]		
*Start* 1418bp	CTGGGTTTTGGTATCCCTGA	ATCGCATATCGAGGCAAGCA
*Gapdh*	CATGACCACAGTCCATGCCATC	TAGCCCAAGATGCCCTTCAGTG
[RACE]		
GSP1		AAACAAAACTGGGGGTGGAG
GSP2		GATGGGTTCATCCTTCCTTG
GSP3		ATGCCCACAAAAGCCATAGA
GSP4	GAAGGCAACCTTCTCTTGCT	
GSP5	CTCGATATGCGATTGGCACC	
AAP	GGCCACGCGTCGACTAGTACGGGIIGGGIIGGGIIG	
AUAP	GGCCACGCGTCGACTAGTAC	
AP	CTGATCTAGAGGTACCGGATCC	
[qRT-PCR]		
*Start*	CCCACTCTTAGCCTCATGGT	CCATCACCCAGCCTGTTCGTT
*Star*	GGGTGGTAGTCAGGAGACAC	TTCACAGCTATGCAGTGGGA
*Cyp11a1*	ATGAGCCAGGAGGTCTAAGC	TCCACCCTATGGGTCCCTAA
*Cyp17a1*	CATGCATGAGAACGGCATCA	CATTCGAACAAGACCAGGGC
*Hsd3b1*	ATGAGCCACTTGTCAACTGGGAGG	TGCTGAAGCCTAAGAACTGAGACTC
*Hsd17b3*	GTCACGATCGGAGCTGAATC	TCGTGTCAGGAGGAATCGTT
*Lhcgr*	CTGTTCACCCAAGACACTCCA	TCAACACCCTAAGGAAGGCA
*Ar*	GATGGTATTTGCCATGGGTTG	GGCTGTACATCCGAGACTTGTG
*Lhb*	ACTGTGCCGGCCTGTCAACG	AGCAGCCGGCAGTACTCGGA
*Gapdh*	TGCACCACCAACTGCTTAGC	GGCATGGACTGTGGTCATGAG
*Aip*	GAGGACGGGATCCAAAAGC	CTGTGCAGCGTCCGAAAGT
[Cloning]		
*Start* full length	GGGTTTTGGTATCCCTGAC	GTTGCCTGGCTTTGGGCATA
[gRNA]		
5’-gRNA	CTAATACGACTCACTATAGAGTACTTCCCTAGGTAAATGGTTTTAGAGCTAGAAATAGCA	
3’-gRNA	CTAATACGACTCACTATAGGTAACATCCTGTCATATTGCGTTTTAGAGCTAGAAATAGCA	
[Genotyping]		
*Start* genotyping	AGACACTGGCCTGATGTTGG	GCTCTAAGATCCTGGCGGTG

### Rapid Amplification of cDNA Ends Analyses

For 5’RACE, cDNA was generated using a gene-specific primer 1 (GSP1) and total RNAs from mouse male germ cells. After purification of cDNA with a QIAquick PCR Purification Kit (Qiagen, Hilden, Germany), oligodeoxycytidine was added by terminal deoxynucleotidyl transferase (Takara). The first-round PCR was performed with an abridged anchor primer (AAP) and GSP2. For the second nested amplification, GSP3 and an abridged universal amplification primer (AUAP) were used.

For 3’RACE, cDNA was prepared by reverse transcription with oligo(dT) connected to an adaptor sequence (AP). The first-round PCR was conducted by using primers, AP and GSP4. The second nested amplification was carried out with the same adaptor primer and GSP5.

All of the PCR products were subcloned into the *Eco*RV site of a pBluescript II KS(+) vector (Stratagene La Jolla, CA, USA) by the TA-cloning method, and 10 subclones for each sample were sequenced. All of the primer sequences are listed in **Table 1**.

### Quantitative RT-PCR

cDNAs were synthesized as described above using the oligo(dT) primer. PCR was performed by using SYBR Green PCR Master Mix for protein-coding genes (Thermo Fisher Scientific) and KOD SYBR qPCR Mix for *Start* (Toyobo). In both cases, the 7300 real-time PCR system (Applied Biosystems, Foster City, CA, USA) was used as previously described (49). The relative expression levels were normalized to endogenous *Gapdh* or *Aip* mRNA and calculated by the comparative Ct method (50). Primer sequences are shown in **Table 1** and **Supplemental Table 1**.

### Preparation of Germ Cell, Sertoli Cell, and Sertoli/Leydig Cell Fractions

Native germ cells were isolated from two adult mouse testes, and their purity was checked by marker gene expression as previously described (46, 49). Sertoli cells were obtained from 11-day-old testes for primary culture as previously described (37, 51). The Sertoli/Leydig cell fraction was obtained from 6-month-old testes by using a discontinuous Percoll gradient (20%, 37%, and 53%) as previously described (44).

### Cytosolic and Nuclear Extraction From Germ Cells

Subcellular fractions were obtained by using NP-40 lysis buffer (10 mM Tris HCl [pH 7.5], 10 mM NaCl, 3 mM MgCl_2_, 0.5% NP 40) containing 1× proteinase inhibitor cocktail (Roche Molecular Biochemicals, Mannheim, Germany) as previously described (44, 52). The nuclear fraction was collected as a pellet after centrifugation, and the supernatant was used as the cytosolic fraction.

### *In Situ* Hybridization

The RNA probes to detect *Start* were prepared as follows. The full length of *Start* was amplified by RT-PCR with mouse testis cDNA and a primer pair of *Start* full length (**Table 1**) using KOD Fx Neo and was subcloned into the *Eco*RV site of a pBluescript II KS+ vector. After the sequence had been confirmed, the full length was cut out by *Eco*RI and *Hin*dIII digestion, blunted by T4 DNA polymerase (Takara), and inserted into the blunted *Pst*I site of a pGEM-3Zf(+) vector. The resulting plasmid was linearized with *Apa*I and *Not*I digestion for synthesizing sense and antisense RNA probes, respectively. Digoxigenin (DIG)-labeled RNA probes were synthesized using a DIG RNA labeling kit (Roche Molecular Biochemicals) with SP6 RNA polymerase for a sense probe and T7 RNA polymerase for an antisense probe.

*In situ* hybridization with the Tyramide Signal Amplification Plus system (PerkinElmer, Waltham, MA, USA) was performed according to the procedure reported previously (44, 53). Briefly, adult mouse testes were fixed for 3 h at 4°C with 4% paraformaldehyde in phosphate buffered saline (PBS). After washing with PBS, each testis was dissected into two pieces with a razor blade under a dissecting microscope. The two pieces of testis were dehydrated, embedded in paraffin, and cut into 7-μm-thick sections. The sections were hybridized with 2 ng/μl of the DIG-labeled RNA probe overnight at 45°C. After washing, the sections were incubated with an anti-DIG-horseradish peroxidase antibody (1:500 dilution; Cat# 1207733, Roche Molecular Biochemicals) for 30 min at room temperature. The reaction with tyramide-Cy3 (1:50 dilution in 1X Plus Amplification Diluent (PerkinElmer), followed by 1:100 dilution with distilled water) was performed at room temperature for 20 min. To detect nuclei, the sections were incubated with 10 μg/ml Hoechst 33258 at room temperature for 10 min. The samples were mounted with a Fluoro-KEEPER Antifade Reagent (Nacalai Tesque, Kyoto, Japan) and observed under an LSM 5 LIVE confocal microscope (Carl Zeiss, Oberkochen, Germany).

### Generation of *Start*-KO Mice

sgRNA sequences were designed by CRISPRdirect (https://crispr.dbcls.jp/) (54). sgRNAs were synthesized and purified by using a CUGA7 gRNA Synthesis Kit (Nippon Gene) according to the manufacturer’s instructions. The selected sequences were *Start* sgRNA-UP (AGTACTTCCCTAGGTAAATGAGG) and *Start* sgRNA-DOWN (GTAACATCCTGTCATATTGCGGG), in which proto-spacer adjacent motif (PAM) sequences are underlined. Oligo DNA sequences used for sgRNA synthesis are listed in **Table 1**.

For injection, oocytes were collected from F1 hybrid (C57BL/6 x DBA/2) BDF1 female mice that were superovulated by standard procedures (55) and fertilized *in vitro* with sperms from male mice of the same genetic background. CAS9 protein (100 ng/µl; Nippon Gene) and sgRNAs (250 ng/µl each) were microinjected into the cytoplasm of fertilized oocytes. The oocytes were cultured in KSOM medium overnight, and embryos at the two-cell stage were transferred to pseudo-pregnant ICR female mice on the next day.

### Genotyping

Tail tips were collected from mice 3-4 weeks after birth and treated with Proteinase K solution (50 mM Tris-HCl, pH 8.0, 100 mM EDTA, 0.5% SDS, and 100 μg/ml proteinase K) at 50°C for 24 hr. After extraction with phenol/chloroform/isoamyl alcohol, genomic DNA was precipitated with isopropanol. Purified DNAs were examined by PCR using a pair of primers listed in **Table 1**.

### Morphological Observation of Testes

Wild-type and *Start*-KO mice were euthanized, and their body weights were measured. Then testes were isolated and their weights were measured. The testes were fixed with Bouin’s solution at room temperature for 2 days, dehydrated, embedded in paraffin, and sectioned at 7 µm in thickness. The sections were stained with hematoxylin and eosin and observed under a light microscope.

### Sperm Count

One cauda epididymis was collected from wild-type and *Start*-KO adult mice, soaked in 0.7 ml of HTF medium (Nippon Medical and Chemical Instruments, Osaka, Japan), and torn into pieces by a sterile 26G needle. After incubation at 37°C with 5% CO_2_ in air for 2 hr, whole medium was transferred into a new tube and mixed with 0.7 ml of 10% neutral buffered formalin (Fujifilm Wako Pure Chemical Corporation, Osaka, Japan) for fixation. The number of sperms was counted manually by using a hemocytometer.

### RNA-Sequencing Analysis

RNA-seq analysis was performed as previously described (36, 56). In brief, total RNAs were isolated from a wild-type testis and *Start*-KO testis (line #2) of mice at the age of 2.5 months using ISOGEN according to the manufacturer’s protocol. The RNAs were treated with TurboDNase to completely remove genome DNA, and the quality of the RNA samples was evaluated by electrophoresis and BioAnalyzer (Agilent Technologies, Palo Alto, CA, USA) with RNA6000 nano tip. cDNA synthesis was performed with 500 ng RNA from each sample by using a TruSeq Stranded mRNA Sample preparation kit (Illumina, San Diego, CA, USA) according to the manufacturer’s protocol. The resulting cDNA library was validated using BioAnalyzer with DNA1000 Chip and quantified using NEBNext Library Quant Kit for Illumina (NEB, Ipswich, MA, USA). A total of 101-cycles single-end sequencing was performed using HiSeq1500 (Illumina) in the rapid mode. After conversion to fastq format, reads were mapped to the mouse reference genome, mm10, with Hisat2 software (v2.1.0) (57). The expression level of each gene was calculated as gene-specific Transcript per Million mapped reads (TPM) using Stringtie (v1.3.3b) (57) with the optional –conservative setting enabled. Total reads, mapping rates, and accession numbers are summarized in **Table 2**.

**Table 2:** Summary of RNA-seq results and accession numbers.

Sample name	Total reads	Overall alignment rate	Accession number
Wild type adult testis (line #2)	28,086,798	95.40%	SRR12700727
*Start*-KO adult testis (line #2)	30,258,284	95.70%	SRR12700726

### Testosterone Measurement

A blood sample from each mouse was collected into a microtube and kept on ice for 1-2 hr until it became clotted. The sample was then centrifuged at 1,000g for 10 min at 4°C. The supernatant was collected as serum and quickly frozen by liquified nitrogen. To measure intratesticular testosterone, tunica albuginea of each adult testis was removed, and the tissue was homogenized in 1 ml of ice-cold PBS. The crude sample was centrifuged at 2,500 rpm for 5 min at 4°C, and the supernatant was collected into a new tube. This centrifugation step was performed twice. The sample was mixed with an equal volume of chloroform, vortexed for 1 min, and centrifuged at 15,000 rpm for 5 min at room temperature. After the centrifugation, the organic phase was collected into a new tube whose cap was removed, and the tube was centrifuged until chloroform was evaporated completely. The precipitate was dissolved in 50 μl methanol and diluted with 350 μl enzyme-linked immunosorbent assay (ELISA) buffer (Cayman Chemical, Ann Arbor, MI, USA). The serum from adult mice was diluted 10 or 20 times with milli-Q water for ELISA analysis, and that from 8-days postpartum (dpp) mice was used after 3.3 times dilution. The intratesticular sample was diluted 100 times with ELISA buffer. Intratesticular and serum testosterone concentration were measured by using a Cayman Testosterone EIA kit according to the manufacturer’s instructions.

### Immunohistochemistry

Paraffin sections of testes were prepared as above at 7 µm in thickness, and de-paraffinized and hydrophilized with xylene, ethanol, and Milli-Q water. The sections were boiled for 45 min with 10 mM sodium citrate-HCl (pH 6.0) and treated with 3% H_2_O_2_-containing PBS. After the H_2_O_2_ treatment, the blocking was performed by soaking the sections with 1% bovine serum albumin in 10 mM Tris-HCl (pH 7.5), 0.88% NaCl, and 0.1% Tween20 for 1 hr at room temperature. An anti-HSD3B antibody (no dilution; Cat# sc-515120, Santa Cruz Biotechnology, Dallas, TX) was reacted with the section for 1 hr at room temperature. After washing with PBS, the sections were subjected to the 2nd antibody reaction for 1 hr at room temperature with a HRP-Labelled Polymer Anti-Mouse (1:4 dilution, Dako). After washing with PBS, the sections were stained with ImmPACT Vector Red (Vector Laboratories, Burlingame, CA) according to the manufacturer’s instructions.

### LH Measurement

Serum samples were collected as described above. Serum LH concentration was measured by using a Luteinizing Hormone ELISA kit (CUSABIO, Wuhan, China) according to the manufacturer’s instructions.

### Plasmid Constructs for Overexpression

The full length of *Start* was amplified and inserted into a pBluescript II KS+ vector as described above. The *Start* sequence was obtained by *Eco*RI and *Hind*III digestion from this vector, and the fragment was blunted by T4 DNA polymerase and inserted into the *Eco*RV site of a pCAG-Hyg vector (Fujifilm Wako Pure Chemicals). Constructs with insertion of the *Start* sequence in both directions were obtained.

### Cell Culture and Transfection

For each of mouse Leydig cell lines, TM3 and MA-10, the hygromycin concentration for selection was preliminarily determined. TM3 cells were cultured in Dulbecco Modified Eagle Medium (DMEM)/F12 containing 2.5% fetal bovine serum, 5% horse serum, 100 U/ml penicillin, 100 µg/ml streptomycin, and 292 µg/ml L-glutamine (Invitrogen, Carlsbad, CA, USA). After coating a 3.5-cm dish with 0.1% gelatin before use, 2.0 × 10^5^ cells were spread in the dish one day before transfection. The next day, 6.0 µl PEI Max (Polyscience, Warrington, PA, USA) was mixed with 200 µl Opti-MEM (Thermo Fisher Scientific) and 2 μg DNA in that order. The mixture was added to the dish after 20-min incubation. Selection was done with the medium containing 250 µg/ml of hygromycin for 8 days.

MA-10 cells were cultured in DMEM/F12 containing 15% horse serum, 100 U/ml penicillin, 100 µg/ml streptomycin, and 292 µg/ml L-glutamine on a dish coated with 0.1% gelatin. One-tenth of confluent cells in a 6-cm dish was spread in a 3.5-cm dish one day before transfection. The next day, 24.0 µl PEI Max was mixed with 200 µl Opti-MEM and 8 μg DNA in that order. The mixture was added to the dish after 20-min incubation. Selection was done with the medium containing 100 µg/ml of hygromycin for 8 days.

### Statistical Analysis

Results are presented as the mean value ± standard deviation (S.D.) of at least three independent experiments. The data were statistically analyzed by Student’s t-test, Welch’s t-test or one-way analysis of variance (ANOVA) followed by the Tukey HSD test. A *P* value less than 0.05 was considered statistically significant. All statistical calculations were done by R software (Ver. 3.5.0; https://cran.ism.ac.jp/bin/windows/).

## Results

### Cloning and Characterization of a Testis-Specific lncRNA, *Start*

**Figure 2:**
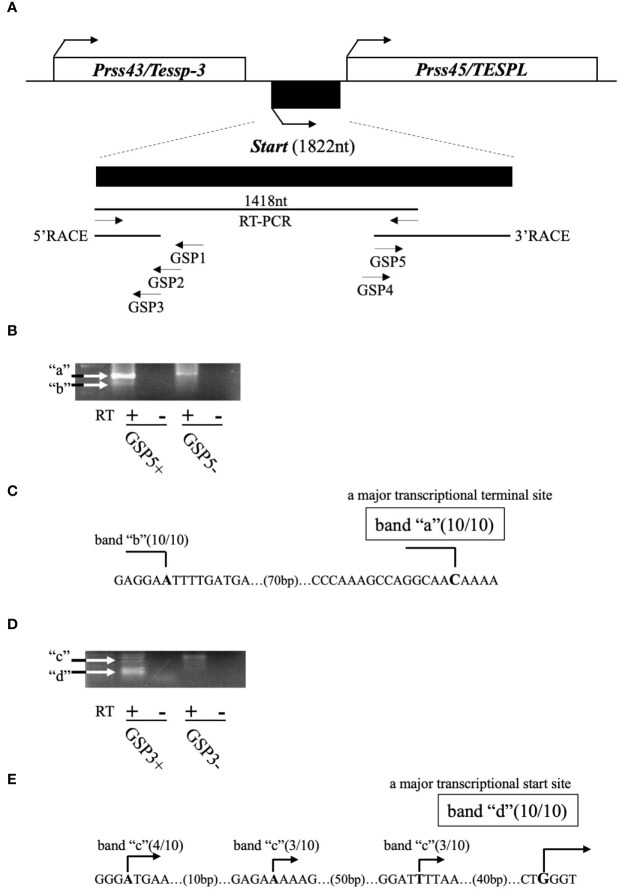
Determination of the full-length sequence of *Start*. **(A)** An overview of RACE analyses. Two protein-coding genes, *Prss43/Tessp-3* and *Prss45/TESPL*, and *Start* between them are drawn as in **Figure 1**. The region transcribed into *Start* is enlarged below the drawing. A 1418-nucleotide sequence that was detected by the first RT-PCR and the sequences determined by 5′ and 3’RACE are indicated by horizontal lines. Positions and directions of three primers (GSP1–GSP3) for 5’RACE and two primers (GSP4 and GSP5) for 3’RACE are also shown by horizontal arrows. **(B)** PCR results of 3’RACE. Two bands (a and b) were obtained by the second PCR as indicated in the gel image. The result of RT-PCR using GSP5 and AP primers is represented as GSP5+, and the result without a GSP5 primer is represented as GSP5-. Reverse transcription was performed with a reverse transcriptase (RT+) or without a reverse transcriptase (RT-). White arrows indicate the specifically amplified bands. **(C)** Transcriptional termination sites of *Start*. Ten subclones from each band (“a” and “b”) were subjected to DNA sequencing, and the number of subclones containing the same transcriptional termination site is indicated on each nucleotide in the genome sequence. Due to its stronger intensity, a cytosine from band “a” was determined to be a major transcriptional termination site. **(D)** PCR results of 5’RACE. Two bands (“c” and “d”) were obtained by the second PCR as indicated in the gel image. Results of RT-PCR using GSP3 and AUAP primers are represented as in **(B)**. **(E)** Transcriptional start sites of *Start*. Ten subclones from each band were subjected to DNA sequencing, and the number of subclones containing each transcriptional start site is shown. Due to its stronger intensity, a guanine from band “d” was determined to be a major transcriptional start site.

Previously, we revealed the presence of two testis-specific lncRNAs at the mouse *Prss/Tessp* locus and we suggested significant roles of these noncoding regions. In this study, we searched for other lncRNAs and found by RT-PCR that a 1418-bp sequence was transcribed in an intergenic region between *Prss43/Tessp-3* and *Prss45/TESPL* genes in the testis (**Figure 2A**). We then determined the 5’- and 3’-ends of this transcript by RACE analyses. In 3’RACE, two bands were specifically amplified by the second PCR (marked as “a” and “b” in **Figure 2B**), and 10 subclones from each were sequenced. It was found that all subclones contained the same sequence for each band. Due to stronger band intensity, a cytosine from band “a” was determined to be a major transcriptional termination site (**Figure 2C**). In 5’RACE, two bands were detected by the second PCR (marked as “c” and “d” in **Figure 2D**), and 10 subclones were checked for each band. For band “c”, three different sequences were detected, but the subclones from band “d” contained a single sequence. Because band “d” was more intense than band “c”, a guanine from band “d” was determined to be a major transcriptional start site (**Figure 2E**). Note that the transcriptional start site was within the primer sequence used for the initial RT-PCR amplifying the 1418-bp product (**Figure 2A**). Consequently, this transcript was defined to be a single-exon structure of a 1822-nucleotide sequence with a poly(A) tail at its 3’ end. Based on the function reported here, we named this transcript *Start*.

**Figure 3:**
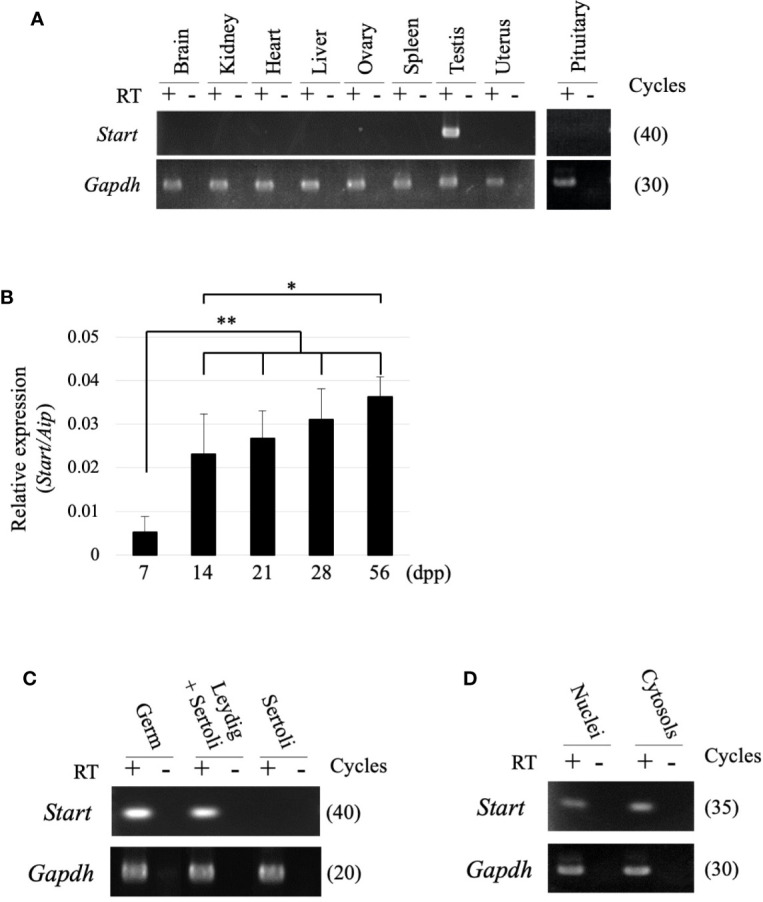
Expression of the *Start* transcript. **(A)** Tissue specificity of *Start*. Total RNAs from nine mouse tissues, as indicated, were collected from adult wild-type mice and used for RT-PCR with a random hexamer for reverse transcription. The *Gapdh* gene was detected as an internal control. The cycle number of each PCR is shown in parenthesis. We obtained similar results with the three biological replicates, and a representative result of the three experiments is shown. **(B)** *Start* expression during testis development. Mouse testes at 7, 14, 21, 28, and 56 dpp were collected, and total RNAs were purified and used for qRT-PCR. The *Aip* gene was used as an internal control to normalize the *Start* expression level. Data are presented as means ± S.D. from three independent experiments with three biological replicates. Statistical significance was analyzed by Tukey’s HSD test. *P < 0.05, **P < 0.01. **(C)** RT-PCR with fractionated testicular cells. Total RNAs from germ cells, Leydig/Sertoli cells, and Sertoli cells were collected and used for RT-PCR. The *Gapdh* gene was utilized as an internal control. The cycle number of each PCR is shown in parenthesis. **(D)** RT-PCR for subcellular localization of *Start*. Total RNAs were purified from nuclear and cytosolic fractions of germ cells and used for RT-PCR. *Gapdh* was used to amplify a region within exon 6 as an internal control. The cycle number is indicated in parenthesis.

RT-PCR was performed using 9 tissues to examine the tissue specificity of *Start*, and a signal was only observed in the testis (**Figure 3A**). During the first wave of spermatogenesis, qRT-PCR showed that *Start* expression was significantly elevated from 7 dpp to 14 dpp and remained at high levels thereafter (**Figure 3B**). We then separated testicular cells into three fractions, germ cells, Leydig/Sertoli cells and Sertoli cells, and performed RT-PCR. As shown in **Figure 3C**, *Start* was expressed in both germ cells and Leydig cells but not in Sertoli cells. To investigate the subcellular localization, germ cells were separated into nuclear and cytosolic fractions. RT-PCR showed more dominant localization in the cytosol, but the signal was also found in the nucleus (**Figure 3D**). These results indicated that *Start* is a germ cell and Leydig cell-specific lncRNA and is activated during sperm development.

**Figure 4:**
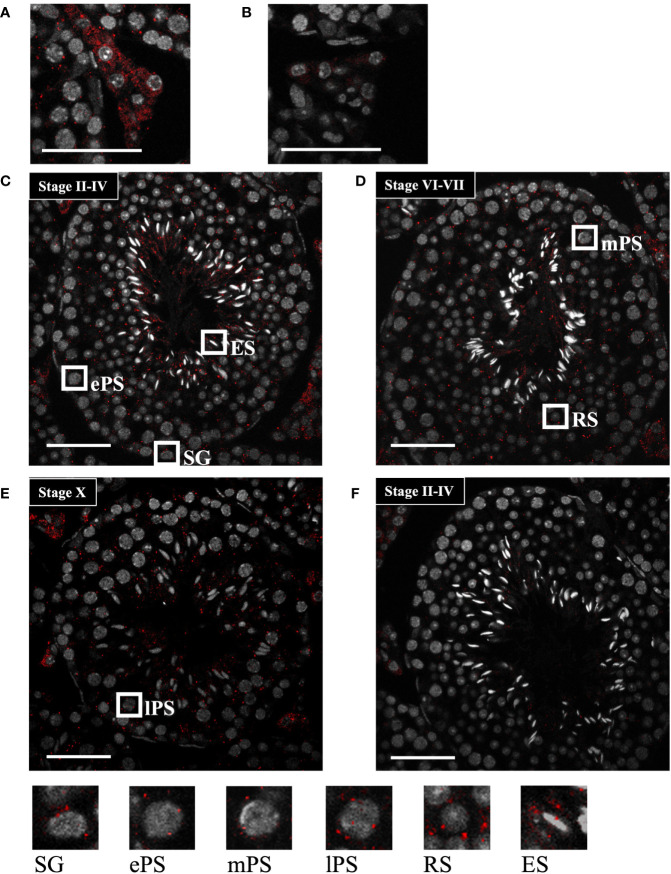
*Start* localization determined by *in situ* hybridization using the tyramide signal amplification system. **(A, B)** Representative images of *Start* localization in interstitial Leydig cells stained with the antisense probe **(A)** and sense probe **(B)**. Staining with the sense probe was performed as a negative control. **(C–E)** Representative images of seminiferous tubules at seminiferous epithelial stages II-IV **(C)**, VI-VII **(D)**, and X **(E)** stained with the antisense probe. The squares indicate the cells enlarged below. As specified in **(C–E)**, the squares emphasize a spermatogonium (SG), primary spermatocytes at the early, mid, and late pachytene stages (ePS, mPS, and lPS, respectively), round spermatid (RS), and elongating spermatid (ES). **(F)** An image of seminiferous tubules at stage II-VI stained with the sense probe as a negative control. In all images, red dots represent *Start* signals, and nuclei were co-stained with Hoechst 33258 (grey). The experiment was repeated twice with different testes, and similar results were obtained. Scale bars, 50 µm.

To further determine the localization of *Start*, we performed *in situ* hybridization by the highly sensitive tyramide signal amplification method (53). Antisense and sense probes for *Start* were hybridized with adult testis sections and the signal was detected as red fluorescence. Consistent with results shown in **Figure 3C**, a strong signal was detected in Leydig cells, mostly in the cytosolic region, by the antisense probe, while few signals were seen in the section hybridized with the sense probe (**Figures 4A, B**). In the seminiferous tubules, many more red dots were observed with the antisense probe (**Figures 4C–E**) than with the sense probe (**Figure 4F**). At seminiferous epithelial stages II-IV and VI-VII (**Figures 4C, D**), the signals were found in all types of spermatogenetic cells including spermatogonia (SG), primary spermatocytes at the early and mid-pachytene stages (ePS and mPS), and round and elongating spermatids (RS and ES, respectively). Consistent with the results of RT-PCR shown in **Figure 3D**, more signals were present in the cytosol of these cell types. At stage X, most of the spermatogenetic cells were stained in a cytosol-predominant manner as in earlier stages (**Figure 4E**), but late pachytene spermatocytes (lPS) appeared to contain more signals in the nuclei than did other types of germ cells. Similar results were obtained with a testis from another mouse. Collectively, the results showed that *Start* was dominantly present in the cytosol of Leydig cells and all types of spermatogenetic cells, though transcripts were also localized in the nucleus. Noticeably, Leydig cells carried the strongest signals among all cell types, suggesting that *Start* plays an important role in Leydig cells.

### Generation of *Start*-Knockout Mice and Morphological Phenotype in Adult Testis

**Figure 5:**
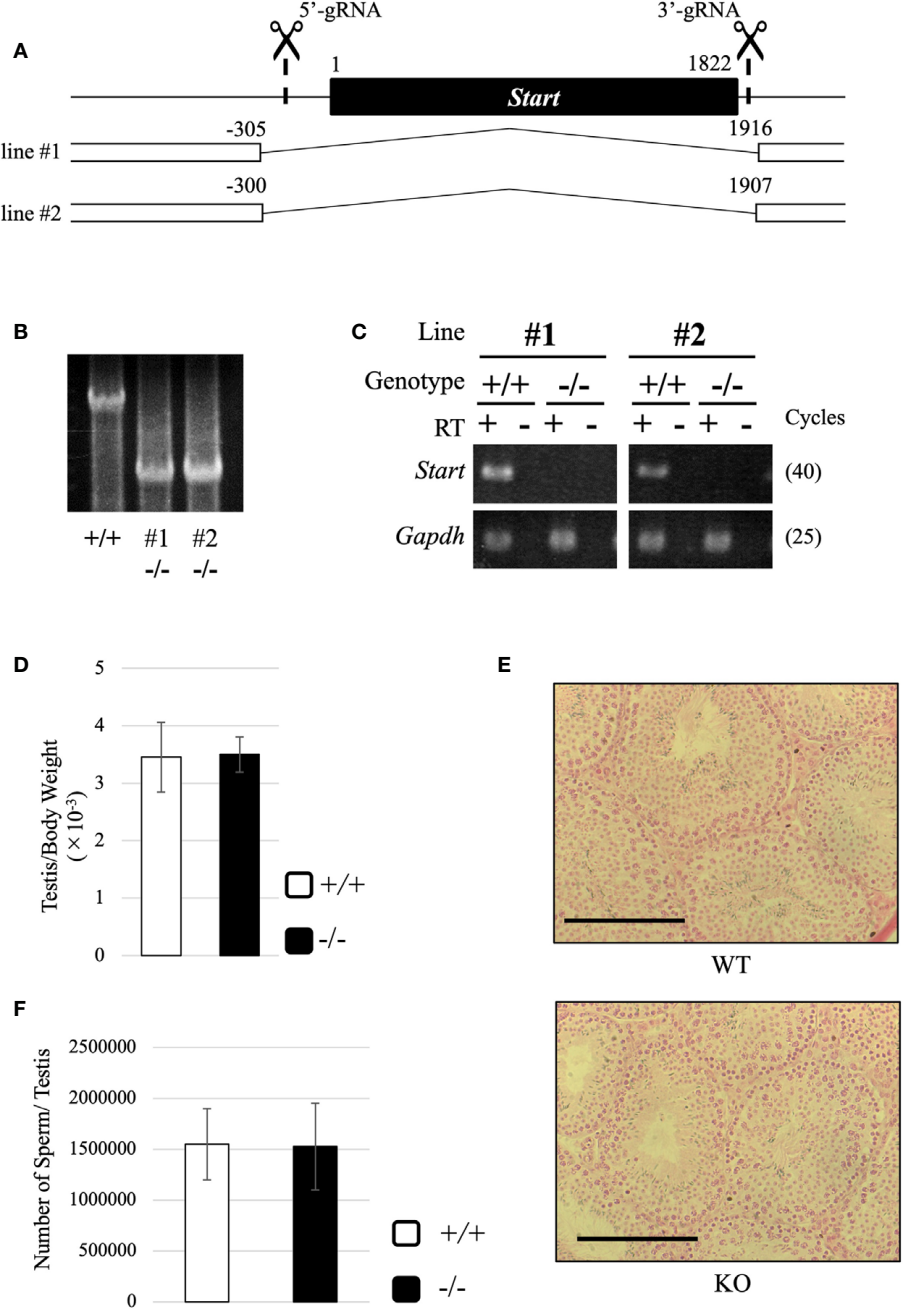
Generation of *Start*-KO mice and morphological characterization of *Start*-KO at 2.5 months. **(A)** Strategy for deletion at the *Start* locus. *Start* is a transcript of 1822 nucleotides in length as shown by a black box. Positions of target sequences of gRNAs are indicated by dashed lines with scissor pictures. By injection of these gRNAs and CAS9 protein into fertilized eggs, two *Start*-KO lines, #1 and #2, were established. The deleted regions of the two KO lines are indicated by the number relative to the transcriptional start site of *Start*. **(B)** A representative result of genomic PCR for genotyping. Genome DNAs from wild-type and *Start*-KO #1 and #2 mice were subjected to PCR amplifying a 4021-bp region including the deleted region. The upper band corresponds to the wild-type locus and the lower band indicates the deleted locus. **(C)** A representative result of RT-PCR for *Start* expression. Total RNAs were collected from adult wild-type and *Start*-KO testes (lines #1 and #2) and used for RT-PCR. The *Gapdh* gene was detected as an internal control. The cycle number of each PCR is shown in parenthesis. **(D)** Ratio of testis to body weight at 2.5 months (lines #1 and #2). Data are presented as means ± S.D. from four independent sets of wild-type and *Start*-KO littermates (four biological replicates). Student’s t-test revealed no significant difference between wild-type and KO mice. **(E)** Testis section at 2.5 months (line #2). Testes from 2.5-month-old wild-type and *Start*-KO littermates were fixed with Bouin’s solution, dehydrated, embedded in paraffin, cut into 7-µm-thick sections, and stained with hematoxylin and eosin. No difference was observed. Scale bars, 100 µm. **(F)** Sperm number at 2.5 months (line #2). Mature sperms were isolated from the cauda epididymis of three sets of 2.5-month-old wild-type and *Start*-KO littermates in HTF medium (three biological replicates). After fixation with 5% formalin, the number was counted. Data are presented as means ± S.D. Student’s t-test showed no significant difference.

To reveal the physiological role of *Start*, we generated *Start*-knockout (KO) mice by the CRISPR/Cas9 system using two guide RNAs designed at the 5’- and 3’-flanking sequences of *Start* (**Figure 5A**). Injection of the guide RNAs and CAS9 protein into fertilized eggs resulted in the generation of four founder mice that had a deletion of *Start*, and two KO lines, lines #1 and #2, were successfully established by mating with wild-type mice. These KO mice contained 2222-bp (#1) and 2208-bp (#2) deletions, which meant that #1 contained 5 and 9 nucleotides longer deletions than #2 at the 5’ and 3’ regions, respectively (**Figures 5A, B**). RT-PCR confirmed no expression of *Start* in these two KO lines (**Figure 5C**). The two lines were used in this study, and all experiments were done with three or more mice except for RNA-seq.

Both #1 and #2 lines of *Start*-KO mice were fertile, and we observed morphology of the adult testis by collecting testes from 2.5-month-old wild-type and *Start*-KO mice. The testis weight normalized to body weight was not significantly different between wild-type and KO mice (**Figure 5D**), and observations of testis sections stained with hematoxylin and eosin did not show any morphological difference (**Figure 5E**). In addition, the number of sperms collected from the cauda epididymides was not changed in KO adult mice (**Figure 5F**). To see whether sex differentiation was affected, we investigated the sex ratio among 16 littermates and we found no significant difference (17 males and 11 females in wild-type vs 22 males and 13 females in *Start*-KO, no significant difference by the chi-square test). Therefore, the adult *Start*-KO testis appeared to be morphologically normal.

### Increased Expression of Testicular Steroidogenic Genes and Decreased Testosterone Level in *Start*-KO Adult Mice

**Figure 6:**
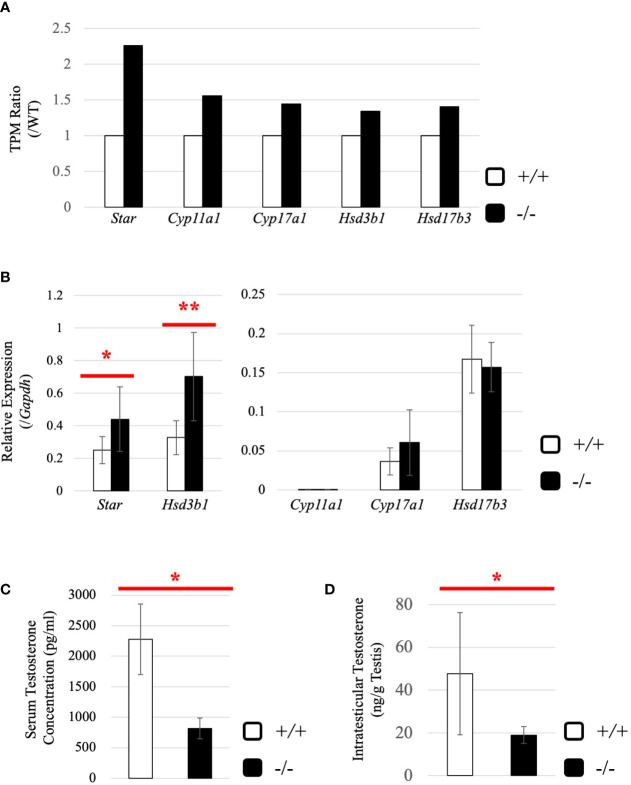
Evaluation of steroidogenic genes and the testosterone level in adults. **(A)** Expression of steroidogenic genes in the testis at 2.5 months (line #2) by RNA-seq. The ratios of TPM values for *Star*, *Cyp11a1*, *Cyp17a1*, *Hsd3b1*, and *Hsd17b3* in a *Start*-KO testis to those in a 2.5-month-old wild-type testis were calculated. The expression level for each gene in the wild-type is set to 1 and fold changes in a KO mouse are presented. **(B)** Expression of steroidogenic genes in the testis at 2.5 months (lines #1 and #2) determined by qRT-PCR. Total RNAs were purified from 2.5-month-old wild-type and *Start*-KO testes and used for qRT-PCR. The *Gapdh* gene was used as an internal control to normalize the expression level of each gene. Data are presented as means ± S.D. from four sets of wild-type and KO littermates (four biological replicates). Statistical significance was analyzed by Welch’s t-test. *P < 0.05, **P < 0.01. **(C)** Serum testosterone level at 2.5 months (line #2). Serum was collected from 2.5-month-old wild-type and *Start*-KO mice, and testosterone concentration (pg/ml) was measured by ELISA. Data are presented as means ± S.D. from three sets of wild-type and KO littermates (three biological replicates). Statistical significance was analyzed by Student’s t-test. *P < 0.05. **(D)** Intratesticular testosterone level at 2.5 months (line #2). Steroids were extracted from 2.5-month-old wild-type and *Start*-KO mice, and testosterone concentration was measured and presented as in **(C)**. Data are presented as means ± S.D. from three sets of wild-type and KO littermates (three biological replicates). Statistical significance was analyzed by Student’s t-test. *P < 0.05.

Subsequently, we attempted to detect differentially expressed genes. Total RNA was purified from whole testes of a 2.5-month-old wild-type and *Start*-KO mice from the same litter and used for RNA-seq analysis. The resultant reads, mapping rate to the mouse cDNA library, and fastq accessions in the NCBI SRA database are summarized in **Table 2**. Interestingly, the *Star* gene encoding a protein to trigger steroidogenesis was found to be upregulated by 2.3 fold in the *Start*-KO testis (**Figure 6A**). Furthermore, expression levels of other steroidogenic genes including *Cyp11a1*, *Cyp17a1*, *Hsd3b1*, and *Hsd17b3* showed 1.3-1.6-fold increases in the *Start*-KO testis (**Figure 6A**). qRT-PCR for these genes using four sets of wild-type and KO mice confirmed that *Star* and *Hsd3b1* genes were significantly upregulated in the *Start*-KO testis (**Figure 6B**). We then measured testosterone concentration in serum and testis. Surprisingly, ELISA analysis showed that the testosterone level was significantly reduced in both serum and testis of KO mice (**Figures 6C, D** **)**. This is inconsistent with the increase of steroidogenic gene expression, but the results suggested that *Start* participates in regulation of the steroidogenesis pathway in Leydig cells.

**Figure 7:**
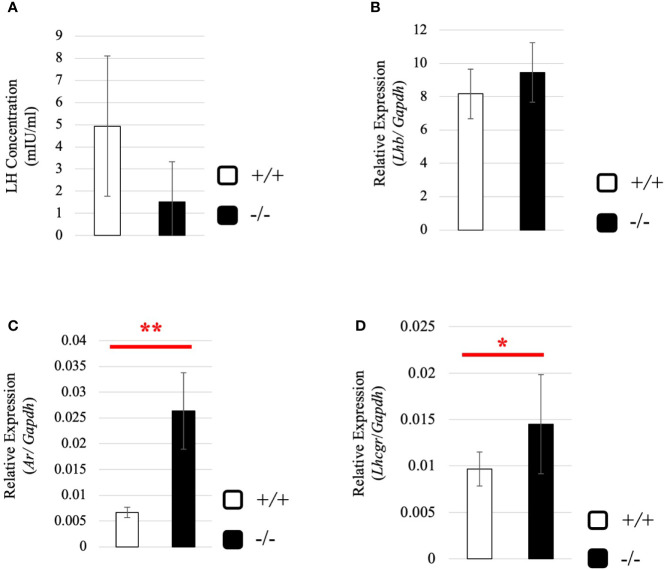
Serum LH level and expression of *Lhb* and hormone receptors in wild-type and *Start*-KO adult mice. **(A)** LH level at 2.5 months (line #2). Serum was collected from 2.5-month-old wild-type and *Start*-KO mice, and LH concentration (mIU/ml) was measured by ELISA using a Luteinizing Hormone ELISA kit (CUSABIO, Wuhan, China). Data are presented as means ± S.D. from three sets of wild-type and KO mice (three biological replicates). Statistical significance was analyzed by Welch’s t-test, but no significance was detected. **(B)** *Lhb* expression in the pituitary at 2.5 months (lines #1 and #2). Total RNAs were purified from pituitaries of 2.5-month-old wild-type and *Start*-KO mice and used for qRT-PCR. The *Gapdh* gene was amplified as an internal control to normalize the expression level. Data are presented as means ± S.D. from four sets of wild-type and KO littermates (four biological replicates). Statistical significance was analyzed by Student’s t-test, but no significance was detected. **(C)** *Ar* expression in the pituitary at 2.5 months (lines #1 and #2). The cDNAs synthesized in **(B)** were used for qRT-PCR to detects *Ar* mRNA. Data are presented as means ± S.D. from three sets of wild-type and KO littermates (three biological replicates), and statistical significance was analyzed by Student’s t-test. **P < 0.01. **(D)** *Lhcgr* expression in the testis at 2.5 months (lines #1 and #2) determined by qRT-PCR. Total RNAs were purified, qRT-PCR was performed, and the expression level was calculated as in **Figure 6B**. Data are presented as means ± S.D. from four sets of wild-type and KO littermates (four biological replicates). Statistical significance was analyzed by Welch’s t-test. *P < 0.05.

To understand why the testosterone level was low despite high expression of steroidogenic genes in adult *Start*-KO testes, we performed several experiments. First, we considered the possibility that conversion into 5α-dihydrotestosterone might be augmented in KO mice. However, qRT-PCR indicated that expression of the *Srd5a1* gene, encoding 5α-reductase, was not changed in *Start*-KO testes (**Supplemental Figure 1**). Second, we investigated the effect of *Start* deficiency on *Androgen receptor* (*Ar*) and found by qRT-PCR that *Ar* expression was not different in wild-type and *Start*-KO testes (**Supplemental Figure 2A**). This suggests that the low level of testosterone might affect the androgen-AR autocrine pathway in adult *Start*-KO testes. Third, we investigated the effect of *Start* deficiency on the HPG axis. In the pituitary, an ELISA assay and qRT-PCR showed that the LH level and *Lhb* mRNA expression were not significantly different in wild-type and *Start*-KO mice (**Figures 7A, B**) but that *Ar* expression was dramatically elevated in KO mice (**Figure 7C**). In the testis, the expression of *Lhcgr*, a receptor of LH, which is supposed to be specific to Leydig cells (58), was significantly increased in *Start*-KO mice (**Figure 7D**). Presumably, the upregulation of *Star* and *Hsd3b1* in adult *Start*-KO testes resulted from a secondary or indirect effect of the *Start* deficiency on the AR autocrine pathway or on the HPG axis (see “*Discussion*”).

### Effect of *Start* Deficiency at a Younger Age

**Figure 8:**
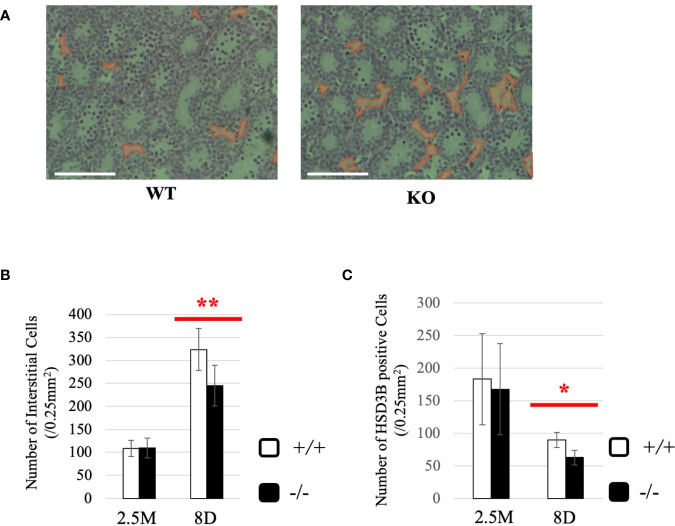
Characterization of *Start*-KO mice at 8 dpp. **(A)** Testis section at 8 dpp (line #1). Testes from 8-day-old wild-type and *Start*-KO littermates were fixed with Bouin’s solution, dehydrated, embedded in paraffin, cut into 7-µm-thick sections, and stained with hematoxylin and eosin. Interstitial regions containing few cells are encircled with red. Scale bar, 100 µm. **(B)** Numbers of interstitial cells at 8 dpp (line #1) and 2.5 months (line #1 and #2). The numbers of interstitial cells in three randomly selected 0.25-mm^2^ squares were counted for each of the three mice. Data are presented as means ± S.D. from average values of three sets of wild-type and *Start*-KO littermates. Statistical significance was analyzed by Student’s t-test. **P < 0.01. **(C)** Numbers of HSD3B-positive cells in wild-type and *Start*-KO testes at 8 dpp (line #1) and 2.5 months (lines #1 and #2). Paraffin sections were prepared and reacted with an anti-HSD3B antibody. The numbers of positively stained cells were counted as in **(B)**. Data are presented as means ± S.D. from average values of three sets of wild-type and *Start*-KO littermates (three biological replicates) for each genotype. Statistical significance was analyzed by Student’s t-test. *P < 0.05.

To gain more insight into effects of *Start* deficiency on steroidogenesis, we investigated *Start*-KO mice at 8 dpp. We first conducted morphological observation by preparing cross-sections of testes of wild-type and *Start*-KO mice from the same litter and staining them with hematoxylin and eosin. As shown in **Figure 8A**, no significant differences were observed within the seminiferous tubules, whereas more interstitial regions containing few cells were observed in the *Start*-KO testis than in the wild-type testis (see regions encircled in red). Indeed, the number of interstitial cells in randomly selected areas (0.25 mm^2^) in the sections was significantly smaller in *Start*-KO testes at 8 dpp than in wild-type testes (**Figure 8B**). In contrast, no significant change was observed in interstitial cells between KO and wild-type mice at 2.5 months of age (**Figure 8B**). Since interstitial cells mainly consist of Leydig cells but also contain small populations of other types of cells (59, 60), we investigated by immunohistochemistry of HSD3B, a marker of Leydig cells (61, 62), whether the altered number of interstitial cells reflected the Leydig cell population. The number of HSD3B-positive cells was significantly decreased in *Start*-KO testes at 8 dpp, while it was not altered at 2.5 months of age (**Figure 8C**). This result was supported by two additional data. First, qRT-PCR indicated a significant decrease of *Insl3* expression, another marker gene for Leydig cells (63, 64), in *Start*-KO testes at 8 dpp but not in adults (**Supplemental Figure 3A**). Second, the expression of *Hsd3b6*, a marker gene for adult Leydig cells (65), showed no difference between wild-type and *Start*-KO testes in adults (**Supplemental Figure 3B**). These results indicated that the number of Leydig cells was decreased in *Start*-KO mice at 8 dpp.

**Figure 9:**
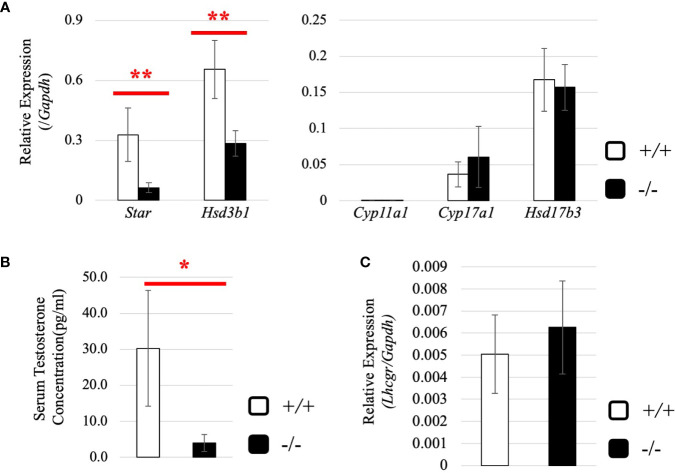
Expression of steroidogenic genes, serum testosterone level, and hormone receptors in the testis at 8 dpp. **(A)** Expression of steroidogenic genes at 8 dpp in the testis determined by qRT-PCR (line #1). Total RNAs were purified from 8-day-old wild-type and *Start*-KO testes and used for qRT-PCR. The *Gapdh* gene was used as an internal control to normalize the expression level of each gene. Data are presented as means ± S.D. from three sets of wild-type and KO littermates (three biological replicates). Statistical significance was analyzed by Welch’s t-test. **P < 0.01. **(B)** Serum testosterone level at 8 dpp (lines #1 and #2). Serum was collected from 8-day-old wild-type and *Start*-KO littermates, and testosterone concentration (pg/ml) was measured by ELISA. Data are presented as means ± S.D. from three sets of littermates (three biological replicates). Statistical significance was analyzed by Welch’s t-test. *P < 0.05. **(C)** *Lhcgr* expression in the testis at 8 dpp. qRT-PCR was performed, and the expression level was calculated as in **(A)**. Statistical significance was analyzed by Student’s t-test, but no significant difference was detected.

Next, we investigated the expression of steroidogenic genes in wild-type and *Start*-KO testes at 8 dpp. Our qRT-PCR indicated that expression levels of *Star* and *Hsd3b1* genes were significantly low in *Start*-KO testes (**Figure 9A**), and serum testosterone level was also found to be lower in *Start*-KO mice than in wild-type mice at 8 dpp (**Figure 9B**). This is in contrast to the results for *Start*-KO mice at 2.5 months of age. To clarify the difference from adults, we examined the expression of hormone receptors in the testis at 8 dpp. While qRT-PCR showed that *Ar* mRNA expression was not different in wild-type and *Start*-KO mice as in adults (**Supplemental Figure 2B**), expression of the *Lhcgr* gene, which was increased in *Start*-KO adult testes (**Figure 7D**), was not changed by *Start* deficiency at 8 dpp (**Figure 9C**). Collectively, the results showed that steroidogenic genes were downregulated in the testis and that the testosterone level was decreased in *Start*-KO mice at 8 dpp.

### Activation of Steroidogenic Genes by *Start* in Leydig Cell Lines

**Figure 10:**
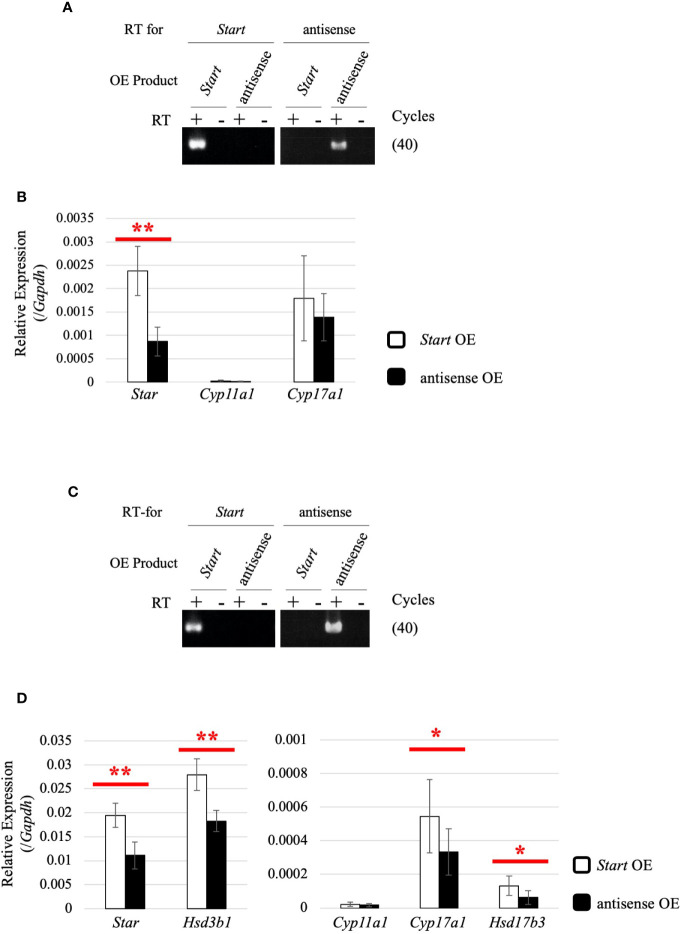
Effect of *Start* overexpression in Leydig cell lines. **(A)** *Start* overexpression in TM3 cells by strand-specific RT-PCR. Total RNAs were purified from TM3 cells that had been transfected with vectors for overexpression of the sense or antisense strand of *Start*. RT-PCR was performed using reverse and forward primers of “*Start* full length” (**Table 1**) for specific reverse transcription of *Start* and its antisense transcript, respectively. ‘RT for’ shows the transcript to be detected by each RT-PCR, and ‘OE Product’ shows the transcript that was overexpressed in the cell. The cycle number of PCR is shown in parenthesis. **(B)** Expression of steroidogenic genes in TM3 cells overexpressing *Start*. Total RNAs purified in **(A)** were used for qRT-PCR using the oligo(dT) primer for reverse transcription. The *Gapdh* gene was used as an internal control to normalize the expression level of each gene. Data are presented as means ± S.D. from three independent experiments (three biological replicates), and statistical significance was analyzed by Welch’s t-test. **P < 0.01. **(C)** *Start* overexpression in MA-10 cells by strand-specific RT-PCR. *Start* overexpression was performed in MA-10 cells. Strand-specific RT-PCR was performed and the results are indicated as above. **(D)** Expression of steroidogenic genes in MA-10 cells overexpressing *Start*. qRT-PCR was performed as in **(B)** and the results are shown as above. Data are presented as means ± S.D. from four independent experiments (four biological replicates), and statistical significance was analyzed by Student’s t-test. *P < 0.05, **P < 0.01.

To determine how *Start* regulates steroidogenic genes in Leydig cells, we utilized two mouse Leydig cell lines, TM3 and MA-10 (66, 67), to perform *Start* overexpression. The antisense sequence of *Start* was overexpressed as a negative control. Strand-specific RT-PCR confirmed successful overexpression of *Start* and its antisense transcript in both cell lines (**Figures 10A, C**). In TM3 cells, *Star* gene expression was significantly increased by *Start* overexpression (**Figure 10B**), and in MA-10 cells, not only *Star* but also *Cyp17a1*, *Hsd3b1*, and *Hsd17b3* genes were significantly upregulated by *Start* (**Figure 10D**). We failed to detect testosterone in the culture media of MA-10 cells overexpressing *Start*, probably due to low capability of testosterone production of this cell line as previously reported (67, 68). These results supported the notion that *Start* acts as an activator of steroidogenic genes in Leydig cells, as shown by results for 8-day-old mice (**Figure 9A**). Based on these results, we concluded that *Start* activates a subset of genes involved in steroidogenesis in Leydig cells.

## Discussion

The current study showed that a testis-specific lncRNA, *Start*, was involved in activation of steroidogenic genes in the testis. As we mentioned in the introduction section, testicular lncRNAs have been mostly evaluated in germ cells, and no *in vivo* evaluation of their functions in Leydig cells has been reported. Thus, this is the first evidence for *in vivo* biological functions of an lncRNA in Leydig cells.

### Possible Function of *Start* in Testicular Germ Cells

While we found a role of *Start* in Leydig cells, it is presumed that *Start* also has some function in germ cells, given its localization in meiotic cells. In general, lncRNAs regulate the expression of their target genes at various levels depending on their subcellular localization (69, 70). *Start* was found to be localized in the cytosol of most germ cells, which raises the possibility that *Start* is related to the regulation of RNA stability or translation of meiotic genes, as was previously reported (71, 72). At the late pachytene stage, *Start* was also localized in the nucleus, suggesting a role in transcriptional gene regulation as was observed for other lncRNAs (73–75). Since expression of various meiotic genes is changed during spermatogenesis (76–78), *Start* may be involved in the regulation of such genes in germ cells.

It is interesting that *Start*, *Tesra*, and *lncRNA-HSVIII*, three lncRNAs at the *Prss/Tessp* locus (**Figure 1**), are all expressed in germ cells and Leydig cells. At the late pachytene stage, when some of the *Prss/Tessp* genes are activated, they are localized in the nucleus. This indicates the possibility that *Prss/Tessp* genes are regulated by these lncRNAs. Indeed, *Tesra* contributes to transcriptional activation of the *Prss42/Tessp-2* gene by collaborating with an enhancer adjacent to *lncRNA-HSVIII* (45). However, the contribution of *lncRNA-HSVIII* to expression of the *Prss42/Tessp-2* gene is not clear, and the function of *Start* in germ cells remains to be elucidated. Therefore, analysis considering the functional relationship of the three lncRNAs at the *Prss/Tessp* locus is necessary to clarify the function of *Start* in germ cells.

### Involvement of *Start* in Development of Fetal Leydig Cells

Our data indicated a decrease in the number of interstitial Leydig cells in *Start*-KO mice at 8 dpp. This was based on the significantly small number of HSD3B-positive cells shown by immunohistochemistry (**Figure 8C**) and a significantly low level of *Insl3* expression shown by qRT-PCR (**Supplemental Figure 3A**). Both changes were observed in *Start*-KO testes at 8 dpp but not in adults. No difference in *Hsd3b6* gene expression between wild-type and *Start*-KO testes supports the notion that *Start* does not affect adult Leydig cells (**Supplemental Figure 3B**). Therefore, we concluded that the number of fetal Leydig cells was decreased by deletion of *Start* at 8 dpp. This suggests the involvement of *Start* in the development of fetal Leydig cells. According to previous studies, fetal and adult Leydig cells are differentiated by distinct regulatory mechanisms (79, 80). However, the mechanism of the differentiation and proliferation of Leydig cells is not fully understood and is even controversial (80). Therefore, *Start* may play some role in the differentiation or proliferation of fetal Leydig cells or their progenitor cells, but the detailed mechanism needs further research. Elucidation of the molecular mechanism will provide an insight into the function of *Start* in the development of fetal Leydig cells.

### Function of *Start* as a Regulator of Steroidogenesis in the Testis

In the present study, we obtained data supporting the involvement of *Start* in the regulation of steroidogenesis in Leydig cells. First, many *Start* transcripts were localized in Leydig cells, where steroid hormones are synthesized (**Figure 4A**). Second, expression of *Star* and *Hsd3b1* genes was changed in *Start*-KO mice at 8 dpp and in adults (**Figures 6B**, **9A**). Third, serum testosterone level was lower in *Start*-KO mice than in wild-type mice both in adults and at 8 dpp (**Figures 6C**, **9B**). Fourth, intratesticular testosterone level was lower in adult *Start*-KO mice than in wild-type mice (**Figure 6D**). Fifth, overexpression of *Start* in two Leydig cell lines significantly increased the expression of steroidogenic genes (**Figure 10**). Although the decreases in the gene expression and testosterone level might be partially due to the reduction in the number of Leydig cells at 8 dpp, these results strongly suggest that *Start* is a regulator of steroidogenesis in the mouse testis by controlling at least *Star* and *Hsd3b1* gene expression in Leydig cells.

Then, how does *Start* regulate the expression of steroidogenic genes? In Leydig cells, *Start* was mostly localized in the cytosol, suggesting its function in post-transcriptional regulation such as mRNA stabilization and translation. In the case of regulation of mRNA stability, lncRNAs are known to form double-strand RNAs with their target mRNAs and induce molecular events leading to RNA degradation or stabilization (81, 82). In translational regulation, lncRNAs generally interact with miRNAs to block their function, resulting in enhancement of the translation of miRNA-targeted mRNAs (75, 83, 84). Given that *Start* is involved in the regulation of multiple target genes and that one of the targets, *Star*, has been reported to be regulated by an miRNA (85), we prefer the possibility of the mechanism involving the miRNA pathway. This may also be one explanation for the reason why *Cyp17a1* and *Hsd17b3* genes were upregulated by *Start* overexpression in MA-10 cells but were not changed in *Start*-KO testes. There might be miRNAs that can regulate *Cyp17a1* and *Hsd17b3* genes and interact with *Start* in MA-10 cells, but they might not be physiologically significant in *Start*-KO testes. Elucidation of the detailed molecular mechanism by which *Start* controls the expression of steroidogenic genes is currently in progress.

It is notable that *Start*-KO mice were fertile despite low levels of testosterone in serum and testis. In general, testosterone production plays a key role in the generation of mature sperm, and many studies have shown that low testosterone levels were associated with male infertility in mammals (86, 87). Meiotic arrest observed in Sertoli cell-specific AR-KO mice supports the notion that testosterone is essential for spermatogenesis (88, 89). However, in some cases, normal progression of spermatogenesis was reported even when testosterone levels were greatly reduced (90, 91), and testosterone levels were not necessarily correlated with fertility (92). These results suggest that a high testosterone level is important for complete spermatogenesis but is not an absolute requirement. In *Start*-KO mice, the reduced level of testosterone might still be sufficient for spermatogenesis.

### Low Testosterone Levels and High Expression Levels of Steroidogenic Genes in Adult *Start*-KO Testes

Interestingly, *Star* and *Hsd3b1* gene expression was upregulated in adult *Start*-KO testes despite the low level of testosterone (**Figure 6**). We do not have a clear explanation for this counterintuitive result, but we have several suggestions based on our data. One possibility is that testosterone is more actively metabolized to other molecules. Although we cannot rule out this possibility, enhanced conversion to 5α-dihydrotestosterone was unlikely considering the results showing no significant change in the expression of *Srd5a1* gene in *Start*-KO testes (**Supplemental Figure 1**). Another possibility is that enhancement of *Star* and *Hsd3b1* expression is caused by autocrine signaling of the androgen-AR trail in Leydig cells. We did not detect any difference in *Ar* expression between wild-type and *Start*-KO adult testes (**Supplemental Figure 2**), but the low level of testosterone might cause a defect in the AR-autocrine pathway. It has been reported that the expression levels of some steroidogenic genes were significantly decreased but that the expression levels of other steroidogenic genes were increased in Leydig cell-specific AR KO testes (93). This indicates that the deficiency of autocrine signaling *via* AR in Leydig cells can cause a disturbance of steroidogenic gene expression. In adult *Start*-KO males, the low level of testosterone might cause a deficiency in this autocrine system and lead to abnormal elevation of *Star* and *Hsd3b1* expression.

There is also the possibility that the low level of testosterone affects the HPG axis in *Start*-KO adults. In the pituitary, a low level of testosterone often causes an increase in LH secretion (94–96), but in *Start*-KO mice, neither LH secretion nor *Lhb* mRNA expression was significantly changed (**Figures 7A, B**). This was probably associated with a dramatic increase in *Ar* expression in the pituitary of *Start*-KO mice (**Figure 7C**), as shown by studies in other tissues (97–99). Notably, *Lhcgr* expression was found to be significantly increased in *Start*-KO adult testes (**Figure 7D**), which is consistent with the upregulation of this receptor gene in AR hypomorphic mutants (100). This was only observed in adults and suggests that the LH signaling pathway in Leydig cells might be enhanced under the condition of normal LH secretion, by which LH-responsive genes such as *Star* and *Hsd3b1* might be upregulated in *Start*-KO adult testes. Given that mouse models overexpressing human chorionic gonadotropin or a constitutively active LH receptor in the testis showed higher levels of testosterone (101–103), the enhancement of the LH pathway might intrinsically cause an increase in testosterone production. Nonetheless, *Start* deficiency caused a significant decrease in testosterone level, suggesting that *Start* is a critical regulator of steroidogenesis in the testis.

At 8 dpp, although an effect of pituitary hormones might remain by minipuberty (104, 105), we verified more direct effects of *Start* deficiency than in adults and observed downregulation of *Star* and *Hsd3b1* genes. Taken together with the increased expression of steroidogenic genes in *Start*-overexpressing TM3 and MA-10 cells (**Figure 10**), the function of *Start* is likely to be enhancement of the expression of steroidogenic genes.

Most previous studies focused on the functions of testis lncRNAs in germ cells (33–36, 41–43, 45, 106) but not on their roles in Leydig cells (40, 107). The results of the present study shed light on the *in vivo* function of *Start*, which plays roles in maintenance of the steroid synthesis pathway in the testis. Thus, this report is the first report on a testis-specific lncRNA that finetunes the steroid synthesis pathway in Leydig cells.

## Data Availability Statement

The raw data supporting the conclusions of this article will be made available by the authors, without undue reservation. The resultant fastq files for RNA-sequencing analysis of a wild-type testis and *Start*-KO testis were deposited in the NCBI SRA database under the accession numbers SRR12700727 and SRR12700726 (BioProject: PRJNA665274). Nucleotide sequence data for *Start* are available in the DDBJ/EMBL/GenBank databases under the accession number LC583804.

## Ethics Statement

The animal study was reviewed and approved by the Institutional Animal Use and Care Committee at Hokkaido University and the Animal Care and Use Committee of the National Research Institute for Child Health and Development.

## Author Contributions

KO and AK designed the study. KO, SM, AS, NT, YS, and MT performed the experiments. KO, SM, AS, NT, YS, TK, HS, and AK analyzed the data. KO, SM, AS, NT, YS, MT, ST, TK, HS, and AK wrote the manuscript. All authors contributed to the article and approved the submitted version.

## Funding

This work was supported by KAKENHI 15H04317 and 20H03285 (AK) and Grant-in-Aid for JSPS Research Fellow 18J21075 (KO) from the Japan Society for the Promotion of Science.

## Conflict of Interest

The authors declare that the research was conducted in the absence of any commercial or financial relationships that could be construed as a potential conflict of interest.

## Abbreviations

Ar, androgen receptor; DIG, digoxigenin; dpp, days postpartum; ELISA, enzyme-linked immunosorbent assay; FSH, follicle-stimulating hormone; GnRH, gonadotropin-releasing hormone; GSP, gene-specific primer; HPG-axis, hypothalamus–pituitary–gonadal axis; KO, knockout; LH, luteinizing hormone; lncRNA, long noncoding RNA; qRT-PCR, quantitative reverse transcription-polymerase chain reaction; RACE, rapid amplification of cDNA ends; RNA-seq, RNA-sequencing; RT-PCR, reverse transcription-polymerase chain reaction; TPM, transcript per million mapped reads.
