# Supplementary material for: A Testis-Specific Long Noncoding RNA, Start, Is a Regulator of Steroidogenesis in Mouse Leydig Cells
Source: Front Endocrinol (Lausanne). 2021 Apr 1;12:665874. doi: 10.3389/fendo.2021.665874 (PMC8061315; doi:10.3389/fendo.2021.665874)
Supplement: Supplementary file 1 [file DataSheet_1.pdf]

Supplemental Figure 1

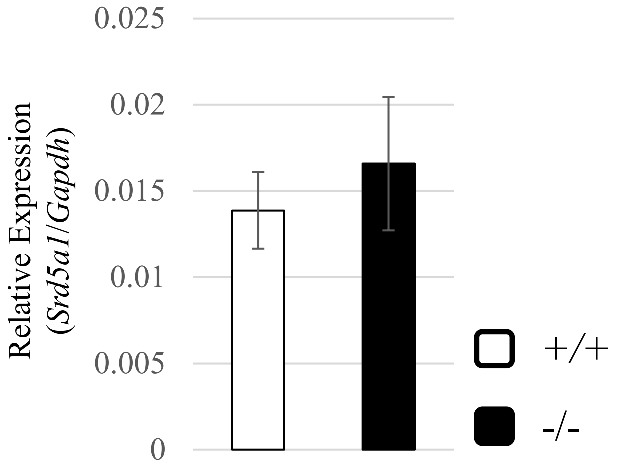

Supplemental Figure 1. Expression of 5α-reductase in the testis at 2.5 months (lines #1 and #2) determined by qRT-PCR. Total RNAs were purified, qRT-PCR was performed, and the expression level of the *Srd5a1* gene was calculated as in Figure 6B. Data are presented as means ± S.D. from four sets of wild-type and KO littermates (four biological replicates). Statistical significance was analyzed by Welch's t-test, but no significant difference was detected.

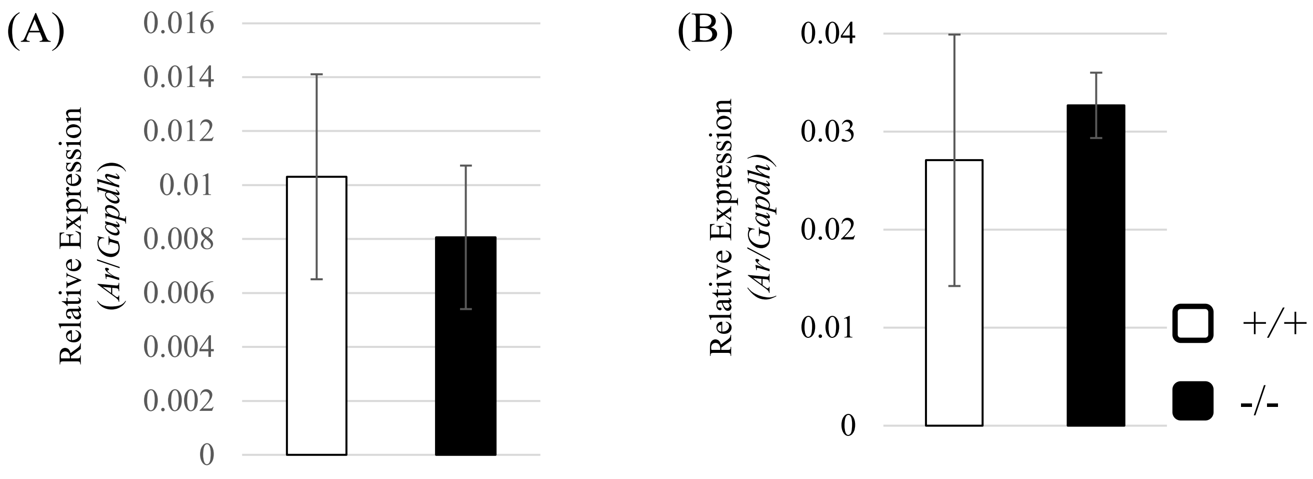

Supplemental Figure 2. Expression of *Ar* in the testis at 2.5 months and 8 dpp determined by qRT-PCR. (A) Total RNAs were purified from wild-type and *Start*-KO mice at 2.5 months (lines #1 and #2), qRT-PCR was performed, and the expression level of the *Ar* gene was calculated as in Fig. 6B. Data are presented as means ± S.D. from four sets of wild-type and KO littermates (four biological replicates). Statistical significance was analyzed by Welch's t-test, but no significant difference was detected. (B) *Ar* expression in wild-type and *Start*-KO testes at 8 dpp (line #1) by qRT-PCR. Data are presented as means ± S.D. from three sets of wild-type and KO littermates (three biological replicates) at 8 dpp. Statistical significance was analyzed by Student's t-test, but no significant difference was detected.

Supplemental Figure 3

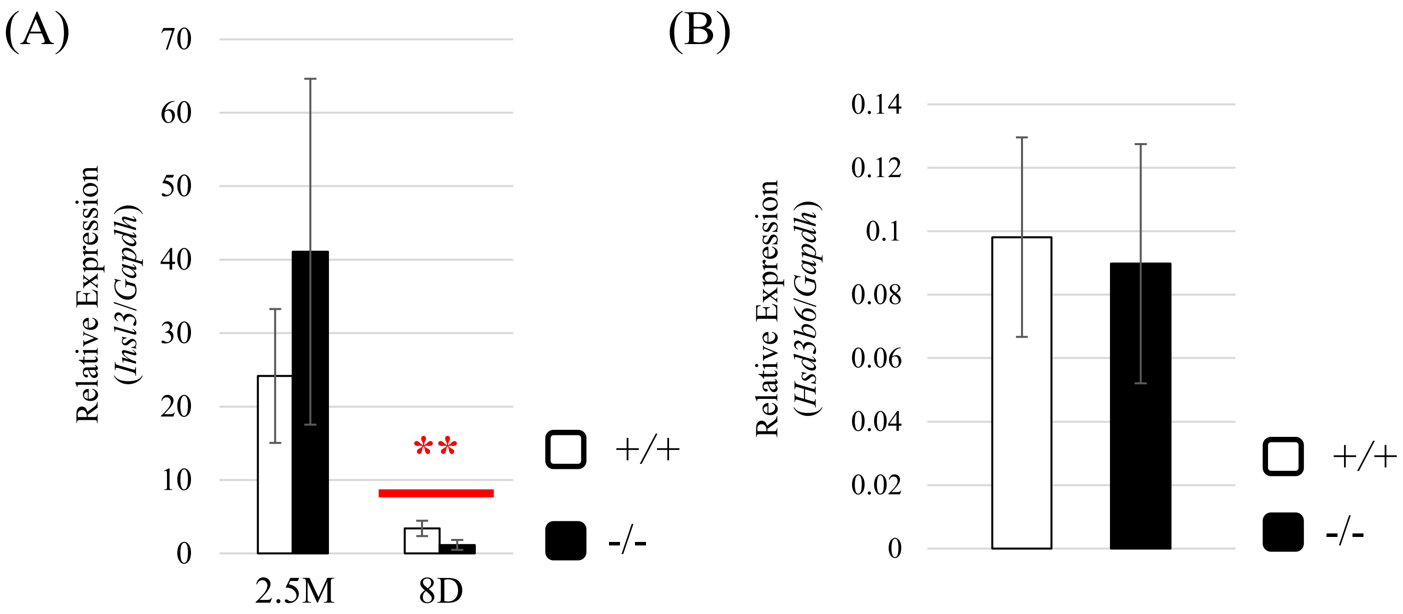

Supplemental Figure 3. Expression of marker genes for Leydig cells in the testis at 2.5 months and 8 dpp determined by qRT-PCR. (A) Expression of *Insl3*, a Leydig cell-specific gene, at 8 dpp (line #1) and 2.5 months (lines #1 and #2). qRT-PCR was performed using cDNAs from wild-type and *Start*-KO testes at the ages of 8 dpp and 2.5 months. Data are presented as means ± S.D. from three sets and four sets of wild-type and KO littermates at 8 dpp and 2.5 months, respectively. Statistical significance was analyzed by Student's t-test. ** $P < 0.01$. (B) Expression of *Hsd3b6*, an adult Leydig cell-specific gene, at 2.5 months (lines #1 and #2) in wild-type and *Start*-KO testes. qRT-PCR was performed, and the expression level was calculated as in (A). Statistical significance was analyzed by Student's t-test, but no significant difference was detected.
