# Supplementary material for: A Testis-Specific Long Noncoding RNA, Start, Is a Regulator of Steroidogenesis in Mouse Leydig Cells
Source: Front Endocrinol (Lausanne). 2021 Apr 1;12:665874. doi: 10.3389/fendo.2021.665874 (PMC8061315; doi:10.3389/fendo.2021.665874)
Supplement: Supplementary file 2 [file Table_1.docx]

**Supplemental TABLE 1** Oligo DNAs used in Supplemental Figures.

| Name | Forward | Reverse |
| --- | --- | --- |
| [qRT-PCR] |  |  |
| *Srd5a1* | GAGGACCACTGGTGAAGAGC | TTTCCTCTCGTGGTGGCATC |
| *Insl3* | CCTGGCTATGTCATTGCAACA | TGGTCCTTGCTTACTGCGATCT |
| *Hsd3b6* | ACCATCCTTCCACAGTTCTAGC | ACAGTGACCCTGGAGATGGT |
